# Decreased Glomerular Filtration Rate Is Associated with Mortality and Cardiovascular Events in Patients with Hypertension: A Prospective Study

**DOI:** 10.1371/journal.pone.0027359

**Published:** 2011-11-11

**Authors:** Rui Zhang, Liqiang Zheng, Zhaoqing Sun, Xingang Zhang, Jue Li, Dayi Hu, Yingxian Sun

**Affiliations:** 1 Department of Epidemiology and Biostatistics, School of Public Health, Peking University Health Science Center, Beijing, People's Republic of China; 2 Library, Shengjing Hospital of China Medical University, Shenyang, People's Republic of China; 3 Department of Cardiology, Shengjing Hospital of China Medical University, Shenyang, People's Republic of China; 4 Department of Cardiology, the First Affiliated Hospital of China Medical University, Shenyang, People's Republic of China; 5 Heart, Lung and Blood Vessel Center, Tongji University, Shanghai, People's Republic of China; Maastricht University, The Netherlands

## Abstract

**Background:**

Few studies reported the associations between decreased glomerular filtration rate (GFR) and mortality, coronary heart disease (CHD), and stroke in hypertensive patients. We aim to assess the associations between GFR and mortality, CHD, and stroke in hypertensive patients and to evaluate whether low GFR can improve the prediction of these outcomes in addition to conventional cardiovascular risk factors.

**Methods and Findings:**

This is an observational prospective study and 3,711 eligible hypertensive patients aged ≥5 years from rural areas of China were used for the present analysis. The associations between eGFR and outcomes, followed by a median of 4.9 years, were evaluated using Cox proportional hazards models adjusting for other potential confounders. Low eGFR was independently associated with risk of all-cause mortality, cardiovascular mortality, and incident stroke [multivariable adjusted hazard ratios (95% confidence intervals) for eGFR <60 ml/min/1.73 m^2^ relative to eGFR ≥90 ml/min/1.73 m^2^ were 1.824 (1.047–3.365), 2.371 (1.109–5.068), and 2.493 (1.193–5.212), respectively]. We found no independent association between eGFR and the risk of CHD. For 4-year all-cause and cardiovascular mortality, integrated discrimination improvement (IDI) was positive when eGFR were added to traditional risk factors (1.51%, *P* = 0.016, and 1.99%, *P* = 0.017, respectively). For stroke and CHD events, net reclassification improvements (NRI) were 5.9% (*P* = 0.012) and 1.8% (*P* = 0.083) for eGFR, respectively.

**Conclusions:**

We have established an inversely independent association between eGFR and all-cause mortality, cardiovascular mortality, and stroke in hypertensive patients in rural areas of China. Further, addition of eGFR significantly improved the prediction of 4-year mortality and stroke over and above that of conventional risk factors. We recommend that eGFR be incorporated into prognostic assessment for patients with hypertension in rural areas of China.

**Limitations:**

We did not have sufficient information on atrial fibrillation to control for the potential covariate. These associations should be further confirmed in future.

## Introduction

The clinical spectrum of chronic kidney disease (CKD) ranges from an unnoticed decrease in glomerular filtration rate (GFR) to end-stage renal failure (ESRD). Individuals with ESRD have a cardiovascular disease (CVD) mortality rate that is 10 to 20 times greater than that in the general population [1], [2], [3]. It has also been demonstrated that decreased GFR is associated with increased all-cause and CVD mortality, incident coronary heart disease (CHD) in general population [1], [4], individuals with manifest arterial disease [5], and high-risk of vascular events [6]. However, few studies reported the associations in patients with hypertension.

Stroke remains the leading cause of disability and second leading cause of death globally. Many factors have been recognized to be independently associated with risk of stroke, which included history of hypertension, current smoking, diabetes mellitus, alcohol intake, atrial fibrillation (AF), and so on. However, it remains uncertain whether reduced GFR constitutes an independent predictor of stroke. In a pooled analysis of community-based studies, estimated GFR <60 ml/min/1.73 m^2^ is not at increased risk of stroke in general population [1]. A recent analysis from PROSPER (Prospective study of Pravastatin in the Elderly at Risk) reported on this association showed a small and nonsignificant increase in stroke risk with decreasing GFR in elderly people [6], and the impact of reduced GFR was shown to be greater for CHD than for stroke in Japanese patients with type 2 diabetes mellitus [7], [8]. In the Rotterdam study, Bos *et al.* [9] highlighted that decreased GFR is a strong risk factor for hemorrhage, but not for ischemic stroke in general population. A large cross-sectional study of older adults showed an association of low GFR with prior stroke [10], however this has not been intensively borne out in prospective cohorts [11].

In summary, in hypertensive patients, there is a relative scarcity evaluating the associations between the reduced GFR with mortality and CHD, especially with stroke in prospective cohorts. Therefore, we selected a representative sample of hypertensive patients in rural areas of China to assess these associations and to evaluate whether low GFR can improve the prediction of these outcomes in addition to conventional CVD risk factors.

## Methods

### Study design and sample

**Figure 1:**
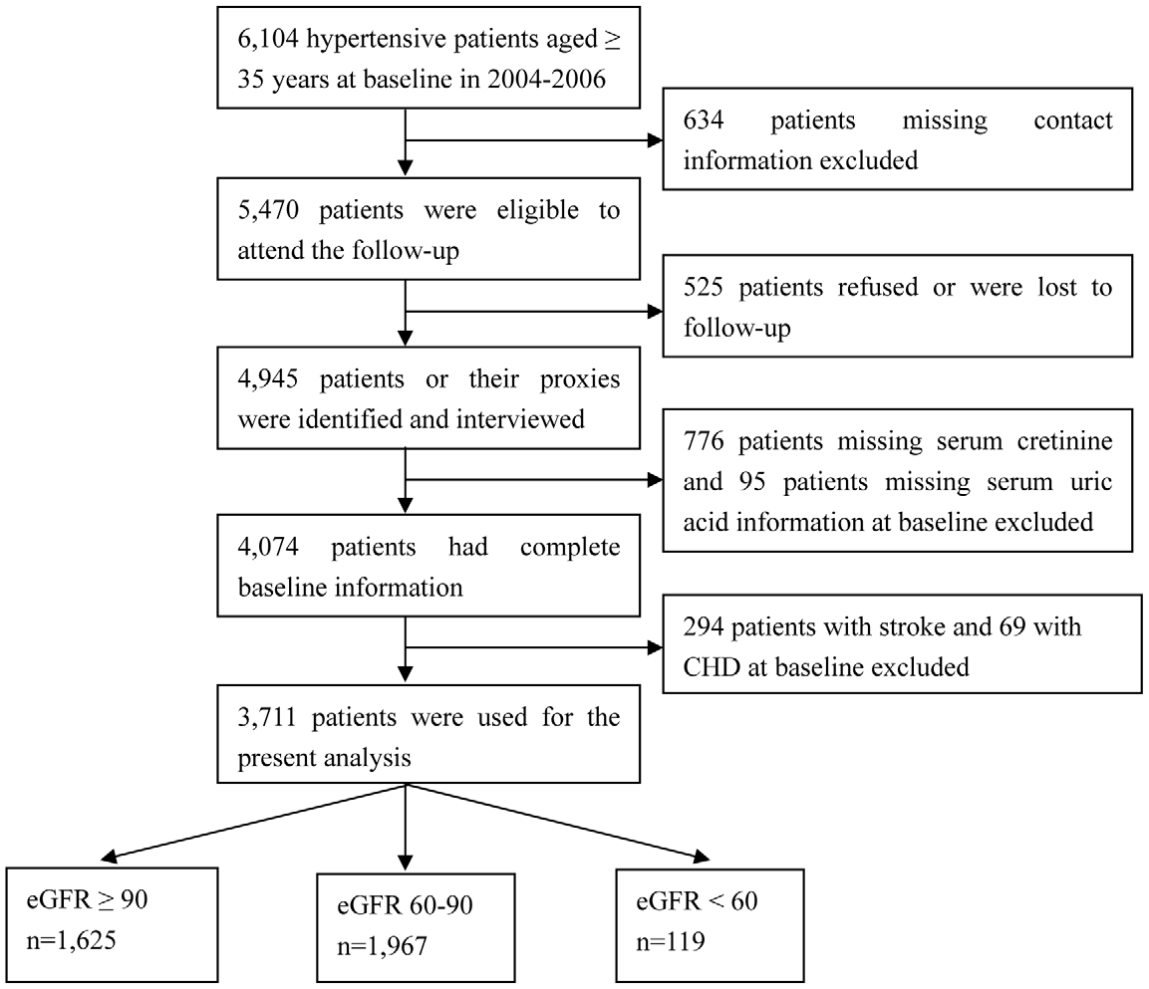
Flow chart of participant recruitment and derivation of the population used in the final analysis.

This is a large-scale epidemiological prospective study. From 2004 to 2006, a multistage, random cluster sampling design was performed to select a representative sample of the rural population with hypertension aged 35 years and older from 50 rural villages of Liaoning Province. The detailed methodology was described elsewhere [12]. In 2010, investigators were invited to participate the follow-up study. Of the 6,104 patients with hypertension at baseline, 634 were not included the follow-up study because study participants' contact information was unavailable. Overall, 5,470 aged ≥35 years at their baseline examination were eligible to participate in the follow-up study. From this population, a total of 4,945 study participants (90.4%) (or their guardians) were identified and agreed to be interviewed as part of the follow-up study. For the present study, hypertensive patients with missing baseline serum creatinine (SCr) (n = 776) and serum uric acid (n = 95), with and having stroke (n = 294) and coronary heart disease (CHD) (n = 69) at baseline were excluded, leaving 3,711 data from hypertensive patients free CVD at baseline for the present analyses. **Figure 1** shows the sample size of patients and exclusion reasons in our study.

### Ethical approval, informed consent and patient privacy

The study complies with the Declaration of Helsinki. China Medical University Research Ethics Committee has approved the research protocol and that written informed consent has been formally obtained from all patients or their guardians. Patients who agreed to participate were explained the content of informed consent which included the purpose of the study, medical items, and confidentiality agreement of personal information. The information about patient's identity was not included with the other data and only the principal investigator had access to this information. No reference to the patient's identity was made at any stage during data analysis or in the paper.

### Glomerular filtration rate assessment

Subjects were asked to fast for at least 12 hours before blood collection. Blood samples were obtained from an antecubital vein into vacutainer tubes containing EDTA. Blood chemical analyses were performed at a central, certified laboratory. SCr was measured by the kinetic alkaline picrate (Jaffé) method on an Olympus AU640 autoanalyzer (Olympus, Kobe, Japan). The GFR was estimated using the equation that originated from the CKD Epidemiology Collaboration (CKD-EPI) equation [13], which is more accurate than the equation form the Modification of Diet in Renal Disease (MDRD) Study group equation [14]. Patients were divided into the following three categories by eatimated GFR (eGFR) at baseline:≥90 (n = 1,625), 60 to 90 (n = 1,967), and <60 (n = 119) ml/min/1.73 m^2^.

### Data collection and physical examinations at baseline

At baseline examination, all participants were recruited and examined at a single clinic visit by their local doctors in their geographical area of origin from 2004 to 2006. There was a central steering committee with a subcommittee for quality control. Before the survey was performed, the research staff had trained the local doctors, which included the purpose of this research, how to administer the questionnaire, the method of measurement, and the study procedures. A strict test was conducted after the training, only those who scored perfectly on the test could become investigators. During data collection, our inspectors had further instructions and support.

Data on demographic variables (age, sex, and ethnicity), smoking status, use of alcohol, information on antihypertensive medications, lipid-lowering drug use, and history of stroke and CHD at baseline were obtained by interviews with a standard epidemiological questionnaire. Drinking status was assessed by alcohol consumption which defined as the weekly consumption of beer, wine and hard liquor converted into grams of alcohol. Alcohol drinker was defined as alcohol consumption at least 8 grams per week during the last year [15]. Current smoking was defined as people who smoked at least one cigarette every day and continued for at least one year. Smoking was assessed as part of the questionnaire. The individuals were asked whether they currently smoked or not (Do you smoke currently?). Body weight and height were measured with subjects wearing light clothing and without shoes according to a standard protocol. History of stroke or CHD at baseline was positive if a stroke or CHD was reported during the baseline interview and confirmed by medical records. Body mass index (BMI) was calculated by the weight in kilograms divided by height in square meters. Diabetes mellitus was defined as fasting serum glucose levels ≥7.0 mmol/l or plasma glucose concentration of ≥11.0 mmol/l2 h after a 75-g oral glucose load or current treatment with insulin or oral hypoglycaemic agents.

Blood Pressure (BP) was measured using a standardized automatic electronic sphygmomanometer (HEM-741C; Omron, Tokyo, Japan). A trained and certified observer used an American Heart Association protocol to perform three BP measurements after study participants had been seated quietly for 5 minutes. Participants were instructed to avoid alcohol consumption, cigarette smoking, coffee/tea, and exercise for at least 30 minutes before these measurements. The mean of 3 BP measures was calculated and used in all analysis. Participants with hypertension at baseline was defined as they had an average systolic BP ≥140 mmHg, and/or an average diastolic BP ≥90 mmHg, and/or use of antihypertensive medication within the previous 2 weeks.

Serum glucose, uric acid, total cholesterol, and high-density lipoprotein cholesterol (HDL-C) were measured in fasting baseline serum with automated enzymatic procedure.

### Follow-up time and outcomes

The follow-up examination was conducted from July to October 2010. Deaths were identified through hospital records and directly contact with their families. Using the International Classification of Diseases, 9th Revision (ICD-9), Clinical Modification [16], deaths due to CVD were assigned a code from 400 through 444 including CHD, stroke, and others. We confirmed that death from CVD on the basis of autopsy reports, death certificates, medical record abstractions, or information obtained from family members.

Stroke was defined as an acute focal neurological deficit lasting longer than 24 hours or resulting in death within 24 hours of the onset of symptoms, and was diagnosed as being due to cerebral lesions of vascular origin. Most stroke cases (n = 167, 90.3%) were diagnosed by computed tomography, MRI including diffusion image, magnetic resonance angiography of the brain and carotid duplex imaging. CHD was defined as myocardial infarction, angina pectoris of which patients received treatment in hospital for ischemic discomfort and diagnose by coronary angiography, coronary revascularization and sudden death. Coronary revascularization was achieved when a patient underwent percutaneous coronary intervention (for example, angioplasty, stenting, atherectomy and laser ablation) or coronary artery bypass graft. The information was obtained by direct reference to medical records by a single investigator.

All materials were independently reviewed by the end-point assessment committee and all members of the end-point assessment committee were blinded to the study participant's baseline risk factor information.

### Statistical analysis

The rates of events are presented as the number of events per 1,000 patient-years, based on the ratio of the number of events observed to the total number of patient-years of exposure up to the terminating event or censor. The rates of events by baseline eGFR subgroups (≥90, 60–90, and <60 ml/min/1.73 m^2^) were tested by Cochran-Armitage Trend Test. We used Cox proportional hazards models to calculate hazard ratios (HRs) with 95% confidence intervals (CI) for the associations between eGFR and incident events. HRs was calculated for eGFR categories, with the eGFR ≥90 ml/min/1.73 m^2^ as the reference group. We constructed 2 models: in model 1, we adjusted for age, sex, and Mongolian ethnicity; and in model 2, we adjusted for age, sex, Mongolian ethnicity, and a propensity score. We calculated the propensity score with a linear regression model entering eGFR as the dependent variable and the independent variables included systolic BP, diastolic BP, pulse rate, BMI, antihypertensive drug use, current smoking, current drinking, diabetes mellitus, serum uric acid, total cholesterol, HDL-C, and lipid-lowering drug use. The proportionality assumption was evaluated by scaled Schoenfeld residuals, and the global fit of the models was evaluated by graphically examining the cumulative hazards function relative to the Cox-Snell residuals.

Furthermore, a logistic regression score was established for 4-year events when we fixed the duration of follow-up to 48 months. With fatal events, stroke, and CHD as the end points, two consecutive logistic models of increasing saturation were performed containing (1) age, sex, Mongolian ethnicity,systolic BP, diastolic BP, pulse rate, BMI, antihypertensive drug use, current smoking, current drinking, diabetes mellitus, serum uric acid, total cholesterol, HDL-C, and lipid-lowering drug use, and (2) eGFR added. To estimate the increase in prediction accuracy, we used the logistic regression score to calculate the area under the receiver-operator characteristic curves (AUC) and compared the AUC based on the models without and with eGFR [17]. We also estimated the integrated discrimination improvement (IDI) (18), which is the difference in an R^2^-like statistic between the traditional and expanded models. Net reclassification improvement (NRI), which examines the net effect of adding a marker to the risk prediction scheme using a statistic described by Pencina et al [18], was computed for stroke and CHD events, but not for all-cause and CVD mortality as no predefined thresholds for low, intermediate, or high risk have been published. The reclassification categories were defined as <1%, 1% to 5%, 5% to 20%, ≥20% for 4-year stroke risk and <5%, 5% to 10%, 10% to 20%, 20% for 4-year CHD risk. Finally, reclassification calibration statistics (RCS) was also calculated to evaluate model calibration. A significant result indicates a lack of fit.

All analyses were performed with SPSS statistical software version 12.0 (SPSS Inc., Chicago, IL, USA). A *P* value less than 0.05 was accepted as indicating statistical significance.

### Results

The median age of the participants of the present study was 56 years (range: 35–91 years) and 2,123 (57.2%) were women. The median of serum creatinine and eGFR were 73.9 µmol/L and 87.6 ml/min/1.73 m^2^, respectively. There were 119 (3.2%) patients with eGFR <60, 1,967 (53.0%) patients with eGFR 60 to 90, and 1,625 (43.8%) patients with eGFR ≥90 ml/min/1.73 m^2^, respectively. Other baseline characteristics are described in **Table 1**. The 2,393 patients who were not included in the present study relative to the included participants (n = 3,711) have the similar age (median: 57 versus 56 years; *P* = 0.225), systolic BP (median: 157 versus 160 mmHg; *P* = 0.507), diastolic BP (median: 96 versus 95 mmHg; *P* = 0.783), and more likely to be female (61.3% versus 57.2%, *P* = 0.002).

**Table 1:** Baseline characteristics of study participants (n = 3,711).

Baseline characteristics	Median* or Percentage	Association with eGFR†	
		β coefficient	*P* value
Age, years	56 (49−64)	−0.810	<0.001
Women, n (%)	2,123, 57.2	−8.156	<0.001
Mongolian, n (%)	752, 20.3	1.226	0.001
Systolic blood pressure, mmHg	160 (146−178)	−0.010	0.269
Diastolic blood pressure, mmHg	95 (89−103)	−0.072	<0.001
Pulse rate, beats/min	75 (69−82)	0.016	0.284
Body mass index, kg/m^2^	23.74 (22.03−25.95)	0.059	0.237
Current smoking, n (%)	1,443, 38.9	0.271	0.491
Alcohol drinking, n (%)	1021, 27.5	1.376	0.003
Diabetes mellitus, n (%)	426, 11.5	−0.898	0.076
Serum uric acid, µmol/L	256.4 (212.2−307.0)	−0.054	<0.001
Total cholesterol, mmol/L	5.18 (4.54−5.84)	−0.017	0.935
HDL-C, mmol/L	1.39 (1.19−1.60)	0.298	0.657
Antihypertensive medication, n (%)	1,446, 39.0	−0.087	0.798
Lipid-lowering drug use, n (%)	101, 2.7	−0.024	0.981
Serum creatinine, µmol/L	73.9 (66.8−82.2)	-	-
eGFR, ml/min/1.73 m^2^	87.6 (78.2−97.0)	-	-
<60, n (%)	119, 3.2	-	-
60–90, n (%)	1,967, 53.0	-	-
≥90, n (%)	1,625, 43.8	-	-

*With 25th and 75th percentiles.

† *β* coefficients and *P* values estimated with linear regression model adjusted for all other characteristics.

HDL-C, high-density lipoprotein cholesterol; eGFR, estimated glomerular filtration rate.

At the time of follow-up examination, 1,446 patients (39.0%) were receiving antihypertensive medications (compound reserpine, n = 763; diuretic, n = 418; calcium antagonist, n = 703; angiotensin-converting enzyme inhibitor, n = 637; and angiotensin receptors antagonist, n = 64). During on median 4.9 years (range: 3.7 to 6.5 years) of follow-up, a total of 202 patients had died and 133 deaths were attributable to CVD. Mortality from all-cause and CVD were 11.47 (95% CI, 9.90 to 13.04) and 7.55 (95% CI, 6.27 to 8.83) per 1,000 patient-years, respectively. Meanwhile, 185 first-ever stroke and 102 first-ever CHD occurred during follow-up and the incident rates of stroke and CHD were 10.75 (95% CI, 9.21 to 12.29) and 5.79 (95% CI, 4.67 to 6.91) per 1,000 patient-years, respectively.

**Figure 2:**
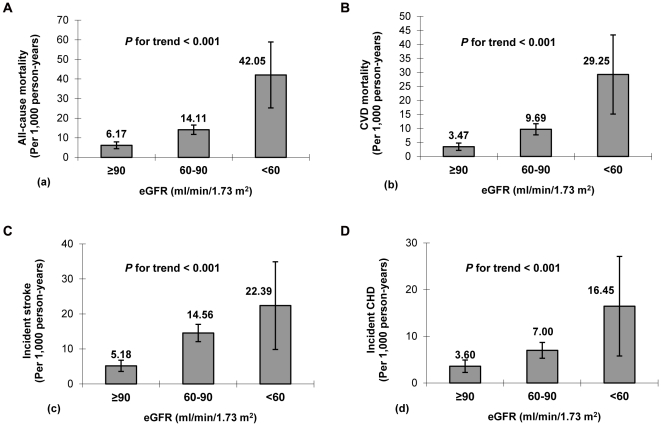
The rates and 95% confidence intervals of all-cause mortality (a), CVD mortality (b), stroke (c), and CHD (d) in patients with hypertension according to eGFR categories. CVD, cardiovascular disease; CHD, coronary heart disease, eGFR, estimated glomerular filtration rate.

**Figure** **2** shows the rates and their 95% CI of all-cause mortality, CVD mortality, incident stroke, and CHD according to eGFR categories. As expected, there was an inverse association between eGFR and incident events (*P* for trend <0.001 each): compared with patients with an eGFR ≥90 ml/min/1.73 m^2^, patients with an eGFR <60 ml/min/1.73 m^2^ had a more than 6-fold increase in all-cause or CVD mortality and a more than 4-fold increase in incident stroke or CHD. After adjustment for sex, age, and Mongolian ethnicity, patients with eGFR <60 ml/min/1.73 m^2^ versus eGFR ≥90 ml/min/1.73 m^2^ had HRs of 2.014 (95% CI, 1.105 to 3.670) for all-cause mortality, 2.784 (95% CI, 1.322 to 5.862) for CVD mortality, 3.127 (95% CI, 1.513 to 6.462) for incident stroke, and 1.917 (95% CI, 0.787 to 4.668) for incident CHD, respectively. Further adjustment for the propensity score of all potential confounders hardly attenuated the HRs. In addition, patients with 60≤ eGFR <90 ml/min/1.73 m^2^ versus eGFR ≥90 ml/min/1.73 m^2^ had a HR of 2.294 (95% CI, 1.532 to 3.437) for incident stroke after adjustment for gender, age, and Mongolian ethnicity and the corresponding HR is 2.047 (95% CI, 1.359 to 3.082) after further adjustment for the propensity score (**Table 2**). There was no evidence of significant interaction between putative confounders and eGFR in predicting each study outcome. No violations of the Cox proportional hazards assumption were observed.

**Table 2:** HRs for the association between eGFR and risk of all-cause mortality, CVD mortality, incident stroke, and incident CHD in patients with hypertension (n = 3,711).

Endpoints	*n,* HR, and *P*−value	eGFR, ml/min/1.73 m^2^			*P* for trend	*P* for eGFR 60–90 *vs.* <60
		≥90 (n = 1,625)	60–90 (n = 1,967)	<60 (n = 119)		
All-cause mortality	*n* (%)	48 (3.0%)	131 (6.7%)	23 (19.3%)		
Model 1*	HR (95%CI), *P*−value	1.000	1.043 (0.703−1.547), 0.834	2.014 (1.105−3.670), 0.022	*P* = 0.073	*P* = 0.016
Model 2†	HR (95%CI), *P*−value	1.000	0.989 (0.663−1.475), 0.956	1.824 (1.047−3.365), 0.044	*P* = 0.145	*P* = 0.032
CVD mortality	*n* (%)	27 (1.7%)	90 (4.6%)	16 (13.4%)		
Model 1*	HR (95%CI), *P*−value	1.000	1.384 (0.837−2.288), 0.205	2.784 (1.322−5.862), 0.007	*P* = 0.013	*P* = 0.027
Model 2†	HR (95%CI), *P*−value	1.000	1.270 (0.763−2.113), 0.358	2.371 (1.109−5.068), 0.026	*P* = 0.042	*P* = 0.059
Incident stroke	*n* (%)	40 (2.5%)	133 (6.8%)	12 (10.1%)		
Model 1*	HR (95%CI), *P*−value	1.000	2.294 (1.532−3.437), <0.001	3.127 (1.513−6.462), 0.002	*P*<0.001	*P* = 0.265
Model 2†	HR (95%CI), *P*−value	1.000	2.047 (1.359−3.082), 0.001	2.493 (1.193−5.212), 0.015	*P* = 0.001	*P* = 0.471
Incident CHD	*n* (%)	28 (1.7%)	65 (3.3%)	9 (7.6%)		
Model 1*	HR (95%CI), *P*−value	1.000	1.147 (0.678−1.939), 0.609	1.917 (0.787−4.668), 0.152	*P* = 0.244	*P* = 0.190
Model 2†	HR (95%CI), *P*−value	1.000	1.024 (0.601−1.744), 0.931	1.530 (0.618−3.787), 0.358	*P* = 0.516	*P* = 0.299

*Model 1: adjusted for age, sex, and Mongolian ethnicity.

† Model 2: adjusted for age, sex, Mongolian ethnicity, and a propensity score (systolic blood pressure, diastolic blood pressure, pulse rate, body mass index, antihypertensive drug use, current smoking, current drinking, diabetes mellitus, serum uric acid, total cholesterol, HDL-C, and lipid-lowering drug use).

HR, hazard ratio; eGFR, estimated glomerular filtration rate; CVD, cardiovascular disease; CHD, coronary heart disease.

When we fixed the duration of follow-up to 4 years, the number of eligible patients were 3,646 [eGFR (ml/min/1.73 m^2^: <60, n = 111; 60–90, n = 1,931; ≥90, n = 1,604]. Of those, there were 147 all-cause deaths, 94 CVD deaths, 135 incident stroke, and 41 incident CHD. With the mortality, stroke, and CHD as the end points, two consecutive logistic models were separately performed. Adding eGFR to the model based on conventional risk factors to predict mortality resulted in positive IDI, whereas improvements in the AUC were no significant (**Table 3**). Upon adding eGFR to the model based on conventional risk factors for 4-year stroke risk assessment, IDI and NRI, but not AUC, indicated discrimination improvement. For stroke risk assessment, RCS yield a chi-square of 13.463 (*P* = 0.019) for the model without eGFR. This value decreased to 10.065 (*P* = 0.073) when eGFR were entered into the model (**Table 3**). For CHD risk assessment, adding eGFR to the model based on conventional risk factors, no discrimination improvement was indicated.

**Table 3:** AUC, IDI, NRI, and RCS for the combined assessment of eGFR and traditional risk factors in predicting mortality, incident stroke, and CHD.

	AUC	IDI	NRI	RCS
**All-cause mortality**				
Model 1*	0.876 (0.854–0.898), *P*<0.001	/	-	-
Model 2†	0.883 (0.862–0.905), *P*<0.001	1.51%	-	-
*P* value *vs.* model 1	0.525	0.016	-	-
**CVD mortality**				
Model 1*	0.873 (0.846–0.900), *P*<0.001	/	-	-
Model 2†	0.885 (0.858–0.911), *P*<0.001	1.99%	-	-
*P* value *vs.* model 1	0.374	0.017	-	-
**Incident stroke**				
Model 1*	0.707 (0.662–0.752), *P*<0.001	/	/	*x* ^2^ = 13.463, *P* = 0.019
Model 2†	0.726 (0.683–0.770), *P*<0.001	0.91%	5.9%	*x* ^2^ = 10.065, *P* = 0.073
*P* value *vs.* model 1	0.399	0.024	0.012	-
**Incident CHD**				
Model 1*	0.887 (0.850–0.925), *P*<0.001	/	/	*x* ^2^ = 2.958, *P* = 0.398
Model 2†	0.891 (0.856–0.926), *P*<0.001	0.30%	1.8%	*x* ^2^ = 2.803, *P* = 0.423
*P* value *vs.* model 1	0.829	0.438	0.823	-

*Variables included age, sex, Mongolian ethnicity, systolic blood pressure, diastolic blood pressure, pulse rate, body mass index, antihypertensive drug use, current smoking, current drinking, diabetes mellitus, serum uric acid, total cholesterol, HDL-C, and lipid-lowering drug use.

† Model 1 + eGFR.

eGFR, estimated glomerular filtration rate; AUC, area under the curve; IDI, integrated discrimination improvement; NRI, net reclassification improvement; RCS, reclassification calibration statistic; CVD, cardiovascular disease; CHD, coronary heart disease.

NRI and RCS computed for incident stroke and CHD only, because the lack of established thresholds of risk for all-cause and CVD mortality.

## Discussion

The present study revealed an inverse association between eGFR and risk of all-cause and CVD mortality, and incident stroke, but not CHD in hypertensive patients in rural areas of China. Compared with the patients with eGFR ≥90 ml/min/1.73 m^2^, patients with 60≤ eGFR <90 ml/min/1.73 m^2^ also carried a substantially increased risk of incident stroke. The predictive value of eGFR remained after adjustment for traditional CVD risk factors. In addition, adding eGFR to the model based on conventional risk factors for 4-year all-cause mortality, CVD mortality, and incident stroke risk assessment indicated discrimination improvement.

In the past, few studies reported the association between eGFR and stroke, and most of these studies have the negative results. In a pooled analysis of 4 population-based studies, participants with CKD was associated with a HR for stroke of 1.17 (95%CI, 0.95 to 1.44; *P* = 0.13) [1]. In another study, the risk of stroke was moderately increased in the lowest GFR quintile compared with the highest GFR quintile with a 92% increased risk among participants with GFR below the 7th percentile. This effect largely disappeared after adjustment for confounding [19]. In our study, there is a strong association between eGFR and stroke in hypertensive patients. The risk of stroke increased by 149.3% among patients with eGFR <60 ml/min/1.73 m^2^, compared with patients with eGFR ≥90 ml/min/1.73 m^2^. Furthermore, the subjects with eGFR 60 to 90 ml/min/1.73 m^2^ also carried a substantial risk of stroke. The predictive value of decreased eGFR remained after adjustment for traditional CVD factors, although some of the risk of CKD is due to its association with traditional vascular factors. To our best of knowledge, this is the first study to obtain the positive association between eGFR and stroke in hypertensive patients in rural areas of China, which is a noteworthy supplement. These different results may be related to the fact that the rate of incident stroke in these studies was lower than in our study.

Mortality and cardiovascular events have strongly been associated with decreased eGFR in community-based studies (subjects considered at low risk) as well as in patients with cardiovascular risk factors (high risk) or in selected patients with established cardiovascular disease (highest risk) [1], [4], [19], [20], [21], [22]. In a pooled analysis of community-based studies, subjects with CKD (eGFR: 15 to 60 ml/min/1.73 m^2^) had a 36% excess risk for all-cause mortality compared with subjects with eGFR ≥60 ml/min/1.73 m^2^ (HR 1.36, 95% CI 1.21–1.53) [1]. Liesbeth Bax *et al*. [5] indicated that the HRs of an estimated GFR ≤60 versus >90 ml/min/1.73 m^2^ were 1.8 (95% CI, 1.2–2.6) for vascular events and 1.4 (95% CI 0.9–2.0) for all-cause death. In a diabetes population, a reduced eGFR was a significant risk factor for CHD [7], [8]. A baseline GFR of less than 53 ml/min/1.73 m^2^ (compared with >104 ml/min/1.73 m^2^) was independently associated with a 32% higher risk for CHD in older high-risk patients with hypertension [23]. Viazzi F *et al* [24] indicated that renal dysfunction is a risk factor for CVD events and all-cause mortality, regardless of traditional confounders, in uncomplicated patients with primary hypertension. In accordance with these results, we found that hypertensive patients with eGFR <60 ml/min/1.73 m^2^ also carried an independently increased risk for all-cause mortality (HR, 1.824) and CVD mortality (HR, 2.371), compared with subjects with eGFR ≥90 ml/min/1.73 m^2^. However, we found no independent association between eGFR and CHD in hypertensive patients in rural areas of China, which is different from previous reports. One possible reason was that the low rate of incident CHD in this rural population and few CHD events accrued, reducing the statistical power of our analyses. In addition, the different duration of follow-up and ethnical groups may partly explain the disparity from other studies.

In most studies, including ours, adjustment for CVD risk factors did not markedly change the associations between eGFR and the risk of adverse outcomes [1], [4], [25], [26], [27], which means either that eGFR is a better marker for vascular pathology than other vascular risk factors or that decreased eGFR is a causal factor in the pathogenesis of mortality and stroke. On the other hand, the adjusted estimates may be underestimations of the true associations because at least part of the presumed effect of impaired renal function on CVD is through the risk factors that were adjusted for.

Multiple explanations exist as to why the decreased eGFR may be associated with an increased risk for mortality and stroke events. GFR is a useful measure of overall kidney function in health and disease. A decrease in kidney function may be associated with an increase in other nontraditional risk factors that were not included in the current analysis, including hyperhomocysteinemia, inflammation, and oxidative stress [28]. In addition, reduced kidney function may be a marker for both duration and severity of other causes of stroke, such as diabetes, and thereby reflect residual confounding from these risk factors. Furthermore, patients with kidney disease likely receive less aggressive therapy for risk-factor modification [17], [26], [29].

It is important to determine whether a decreased eGFR provides additional risk information over and above the assessment of conventional cardiovascular risk factors. In the present study, consecutive multiple regression models and the measurements of discrimination improvement revealed that upon adding eGFR into the model based on conventional cardiovascular risk factors resulted in a significant improvement in its predictive value of 4-year risk of mortality and stroke. In our study, adding eGFR to model based conventional risk factors resulted in 5.9% of patients were reclassified into new stroke risk categories, which may benefit the most by being placed into a higher risk category for intervention with secondary prevention measures.

Some limitations should also be considered in light of these results. Firstly, the limitation of the study is the small number of patients with an eGFR <60 ml/min/1.73 m^2^ (n = 119, 3.2%) and as a consequence, the number of events are very small, which may induce the possible imprecise estimates and limited external validity of findings. Secondly, the study subjects derived from a previous study in rural areas of China and the lack of almost 20% of baseline creatinin levels may implicate some selection bias. Our results may not extrapolate to other populations, and further study is warranted in more ethnically diverse populations. Thirdly, serum creatinine is influenced by nonrenal factors and additional measurement of urinary albumin might intermediate the causal role of eGFR in adverse CVD outcomes. However, urinary albumin was not measured in our study. Finally, AF is an independent risk factor for stroke. It is associated with 4–5-fold higher risk than in the unaffected population [30]. Also, 15% of all strokes and 25% of strokes in patients >80 years old are attributable to AF. In addition, AF is one modifiable risk factor for stroke; it is strongly associated with an elevation of stroke risk that can be greatly diminished using antithrombotic therapy. However, we did not have sufficient information on concomitant AF or concomitant oral anticoagulation/ASS therapy to control for these potential covariates. Further study controlling for the potential covariates (AF and anticoagulation/ASS therapy) to evaluate the eGFR and stroke is encouraged in future.

In conclusion, the decreased eGFR is an independent risk factor for adverse mortality, CHD, and stroke in patients with hypertension in rural areas of China. The presence of vascular disease and other risk factors did not alter the results. Addition of eGFR significantly improved the prediction of 4-year mortality and incident stroke over and above that of conventional risk factors. We strongly recommend that eGFR be incorporated into prognostic assessment of patients with hypertension in rural areas of China.
